# Viral Kinetics Suggests a Reconciliation of the Disparate Observations of the Modulation of Claudin-1 Expression on Cells Exposed to Hepatitis C Virus

**DOI:** 10.1371/journal.pone.0036107

**Published:** 2012-04-24

**Authors:** Pranesh Padmanabhan, Narendra M. Dixit

**Affiliations:** Department of Chemical Engineering, Indian Institute of Science, Bangalore, India; National Research Council of Italy (CNR), Italy

## Abstract

The tight junction protein claudin-1 (CLDN1) is necessary for hepatitis C virus (HCV) entry into target cells. Recent studies have made disparate observations of the modulation of the expression of CLDN1 on cells following infection by HCV. In one study, the mean CLDN1 expression on cells exposed to HCV declined, whereas in another study HCV infected cells showed increased CLDN1 expression compared to uninfected cells. Consequently, the role of HCV in modulating CLDN1 expression, and hence the frequency of cellular superinfection, remains unclear. Here, we present a possible reconciliation of these disparate observations. We hypothesized that viral kinetics and not necessarily HCV-induced receptor modulation underlies these disparate observations. To test this hypothesis, we constructed a mathematical model of viral kinetics *in vitro* that mimicked the above experiments. Model predictions provided good fits to the observed evolution of the distribution of CLDN1 expression on cells following exposure to HCV. Cells with higher CLDN1 expression were preferentially infected and outgrown by cells with lower CLDN1 expression, resulting in a decline of the mean CLDN1 expression with time. At the same time, because the susceptibility of cells to infection increased with CLDN1 expression, infected cells tended to have higher CLDN1 expression on average than uninfected cells. Our study thus presents an explanation of the disparate observations of CLDN1 expression following HCV infection and points to the importance of considering viral kinetics in future studies of receptor expression on cells exposed to HCV.

## Introduction

HCV entry into target cells is a multi-step process involving interactions of the viral envelope proteins E1 and E2 with several cell surface receptors, namely, scavenger receptor class B type I (SR-BI) [1], the tetraspanin CD81 [2], [3], and the tight junction proteins claudin-1 (CLDN1) [4] and occludin [5] (reviewed in [6]). Recent studies suggest a role of the CD81- CLDN1 receptor complex in HCV entry [7]–[9]. Antibodies targeting the CD81-CLDN1 interaction effectively blocked the entry of HCV, including that of escape variants from patient sera [10], [11]. The host cofactors epidermal growth factor receptor (EGFR) and ephrin receptor A2 (EphA2) mediate HCV entry through regulation of the CD81-CLDN1 association [12]. CLDN1 also appears to be necessary for cell-cell transmission of infection [13], [14]. Modulation of the expression of CLDN1 on cells is expected therefore to alter the susceptibility of cells to HCV infection.

Two recent studies examined CLDN1 expression on cells exposed to HCV and made conflicting observations. Reynolds *et al.* [15] found a significant increase in CLDN1 expression on infected cells compared to uninfected cells, suggesting up-regulation of CLDN1 after infection. In contrast, Liu *et al.* [16] found that the mean CLDN1 expression on cells decreased following HCV infection, suggesting down-modulation of CLDN1 after infection. The latter observation suggests a role for HCV-mediated CLDN1 down-modulation in superinfection exclusion. Superinfection exclusion is the process by which a cell once infected becomes resistant to further infections, and has been observed with HCV *in vitro* [16]–[18]. Cellular superinfection has important implications in viral evolution via recombination and may affect disease progression and the emergence of resistance to direct acting antiviral agents [19], as observed with HIV [20]–[22]. The conflicting observations above leave unclear the influence of HCV infection on CLDN1 expression and hence its role in superinfection exclusion. Here, we present a possible reconciliation of these conflicting observations.

We recognize that different cells in culture express different levels of CLDN1 and may consequently be susceptible to HCV infection to different extents. Cells with high CLDN1 expression may be more readily infected than cells with low CLDN1 expression. At the same time, cells with high CLDN1 expression may be outgrown by cells with low CLDN1 expression as the latter cells may remain uninfected and proliferate unhindered by HCV. The underlying viral kinetics may therefore skew the distribution of CLDN1 expression on cells following exposure to HCV and may explain the above conflicting observations. To test this hypothesis, we employed a mathematical model of viral kinetics.

Mathematical models of HCV kinetics and drug therapy have provided valuable insights into disease pathogenesis and treatment outcomes [19], [23]–[36]. Recently, we constructed a mathematical model of viral kinetics in vitro that quantitatively described several independent observations of the dependence of HCV entry and kinetics on the expression of CD81 on target cells and estimated the minimum number of E2-CD81 complexes necessary for HCV infection [37]. Here, we adapted our model to describe experiments where cells with a known distribution of CLDN1 expression were exposed in culture to HCV and the resulting changes in the distribution measured. We found, in agreement with Reynolds *et al.* [15], that the preferential infection of cells expressing high CLDN1 ensured that measurements yielded higher CLDN1 expression on infected cells than uninfected cells. At the same time, retarded growth of infected cells compared to uninfected cells manifested as a shift of the overall CLDN1 expression in culture to lower levels as observed by Liu *et al.* [16]. Further, our model, without requiring active modulation of CLDN1 by HCV, provided good fits to the latter data, suggesting that the role of HCV-induced modulation of CLDN1 expression in superinfection exclusion may require further examination.

## Results

### Model formulation

**Figure 1:**
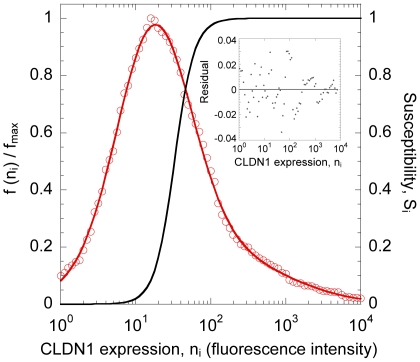
Expression of CLDN1 on cells and their susceptibility to infection. Best-fit (red line) of the normalized distribution of CLDN1 expression, 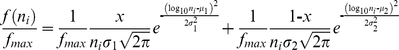, where 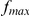 is the maximum value of 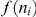, to experimental data [16] (red circles). The best-fit parameter estimates (95% CI) are 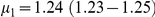, 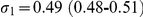, 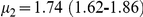, 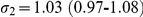, 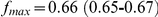, and 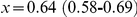. The corresponding susceptibility, 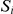, as a function of CLDN1 expression is also shown (black line). (Inset) Residuals (symbols) of the best-fit to experimental data; the mean error is 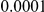 and is not significantly different from zero (line) (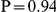 using the two tailed t-test).

We considered *in vitro* experiments where a population of target cells, 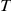, with a known distribution of the CLDN1 expression level was exposed to HCVcc (cell culture adapted) virions, 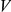. The distribution of CLDN1 expression on the target cells was well described by a mixture of two log-normal distributions (Fig. 1). We divided the target cells into different subpopulations 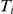 with distinct CLDN1 expression levels 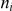, where 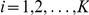. We let the dependence of the relative susceptibility of cells 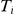 to infection, 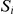, on the CLDN1 expression, 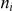, be characterized by a Hill function (Methods). Accordingly, 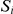 increased with 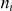 (Fig. 1). Above a certain 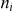 (≈20 fluorescence units), CLDN1 expression did not limit entry and cells were nearly completely susceptible (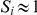). Below a certain 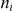 (≈5 fluorescence units), cells remained refractory to infection (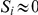). Following exposure to HCV, cells 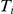 were assumed to proliferate, die, and be infected at a rate proportional to 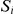, yielding infected cells 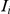. Infected cells were lost at an enhanced rate compared to uninfected cells due to virus-induced cytopathicity *in vitro* [38], [39]. Free virions were produced by infected cells and were cleared. We constructed dynamical equations to describe the ensuing viral kinetics (Methods), which we solved using parameter values representative of HCVcc infection *in vitro* (Table 1).

**Table 1:** Summary of model parameters and their values employed.

Parameter	Description	Value (95% CI)	Source
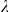	Proliferation rate constant of target cells	0.44 d^−1^	[37]
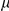	Death rate constant of target cells	1.7×10^−4^ d^−1^	[37]
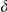	Death rate constant of infected cells	1.1×10^−2^ d^−1^	[37]
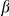	Infection rate constant of cells with excess CLDN1 expression	1.2×10^−4^ ml•(ffu•d)^−1^	[37]
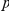	Viral production rate per infected cell	2.78 ffu•(ml•d)^−1^	[37]
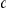	Virion clearance rate constant	23.2 d^−1^	[37]
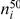	CLDN1 expression level at which 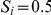	33 (31–35) fluorescence units	Best-fit (Fig. 3)
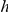	Hill coefficient	3.3 (3.0–3.6)	Best-fit (Fig. 3)

### Dependence of viral kinetics on CLDN1 expression

**Figure 2:**
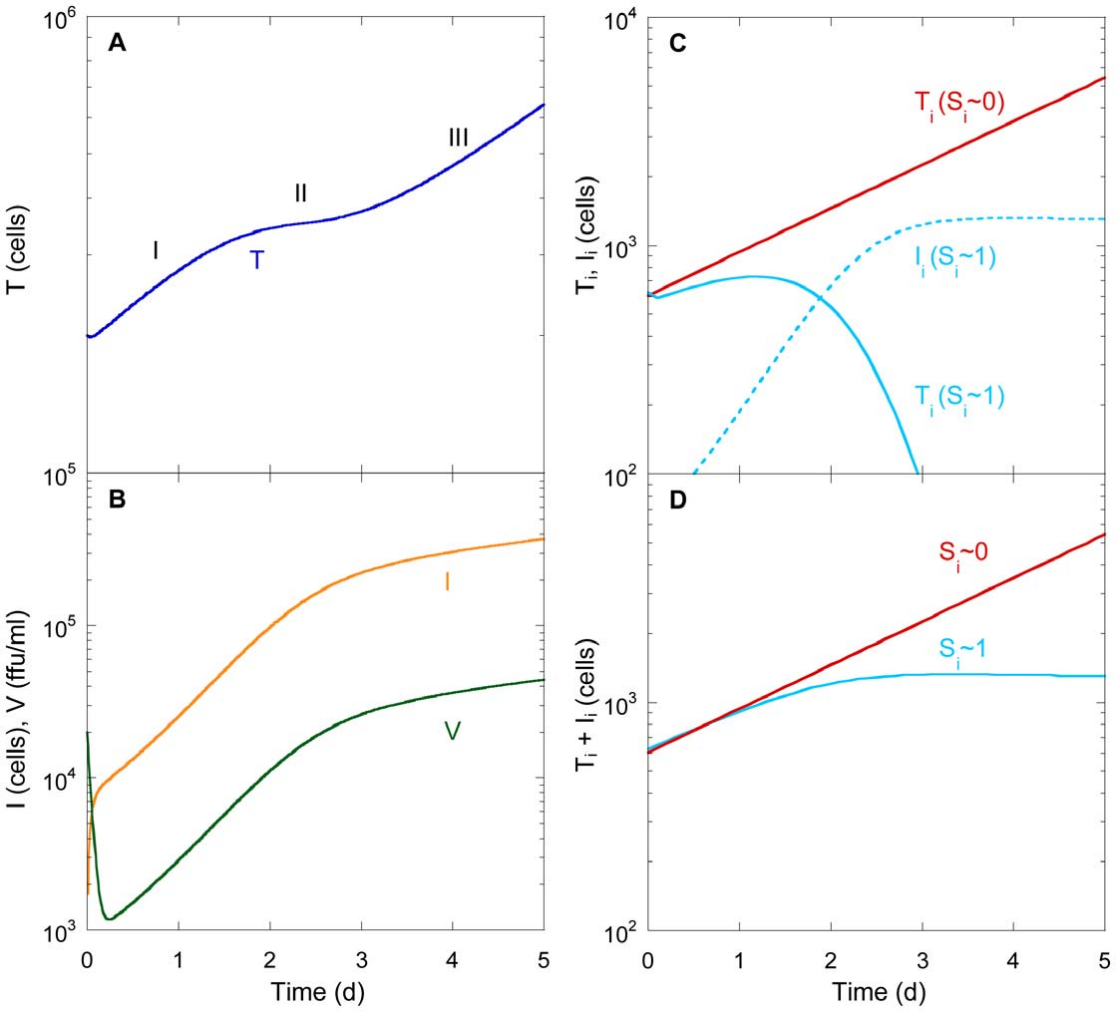
Model predictions of HCV viral kinetics in vitro. The time evolution of (A) uninfected cells, 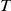, (B) infected cells, 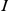, and viral load, 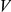, (C) uninfected cell subpopulations, 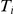 (solid line), and infected cell subpopulations, 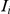 (dashed line), corresponding to 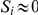 (red) and 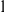 (cyan) and (D) total subpopulations, 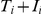, corresponding to 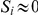 (red) and 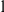 (cyan). The three phases of infection are marked in (A). Initial conditions: 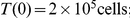 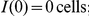 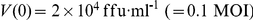. Parameters employed are listed in Table 1.

We found that infection proceeded in three phases (Fig. 2A). In the first phase, the population of uninfected cells, 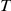, rose because of cell proliferation. Simultaneously, infection of cells by virions resulted in the growth of infected cells, 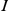 (Fig. 2B). The viral titre, 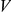, declined initially as viral clearance dominated viral production from the small population 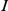 (Fig. 2B). As 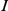 grew, viral production increased and compensated viral clearance. 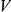 then evolved proportionally to 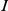, indicating the establishment of a pseudo-steady state between viral production and clearance (Fig. 2B). The growth of 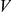 increased the rate of infection of 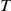; the loss of 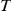 due to infection then became comparable to its growth by proliferation, so that, in the second phase, 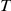 exhibited a plateau (Fig. 2A). Not all cells, however, were equally susceptible to infection. For subpopulations with high CLDN1 expression (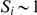), infection dominated proliferation. Accordingly, 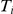 for such subpopulations declined leaving behind infected cells, 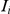 (see 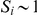 in Fig. 2C). The total subpopulation 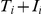 thus reached a plateau (Fig. 2D). In contrast, for subpopulations with low CLDN1 expression, proliferation dominated infection so that 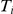 continued to grow (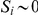 in Fig. 2C) and the total subpopulation 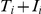 continuously increased (Fig. 2D). Thus, in the third phase, 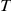 rose again (Fig. 2A) due to the proliferation of the latter cells. 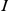 (and hence 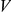) remained nearly constant in the third phase (Fig. 2B) as new infections occurred rarely and the lifespan of infected cells (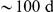) was much larger than the duration of the experiments (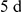). This triphasic kinetics was analogous to the predictions of our previous model and corresponding experiments where HCV infection was limited by CD81 expression [37]. We examined next whether this kinetics could reconcile observations of CLDN1 expression on cells following exposure to HCV.

### Time-evolution of the distribution of CLDN1 expression

**Figure 3:**
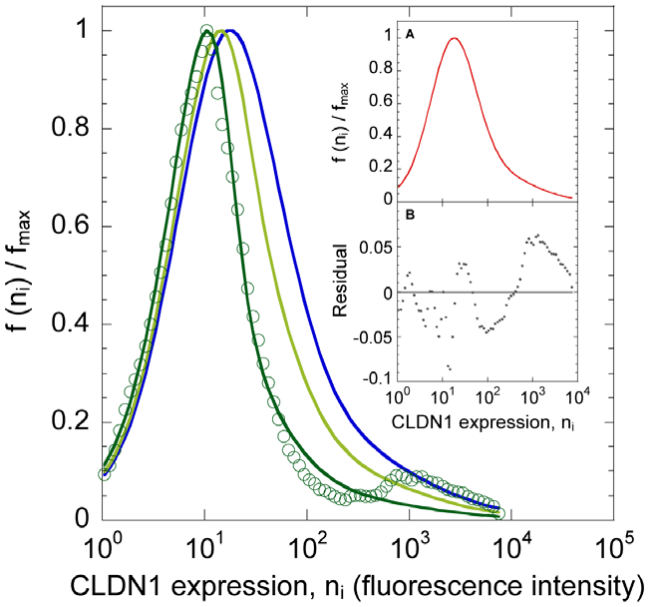
Evolution of the distribution of CLDN1 expression. Model predictions (lines) of the normalized distribution of the CLDN1 expression on cells at day 1 (blue), day 3 (light green), and at day 5 (dark green) post-infection, the latter fit to corresponding experimental data [16] (green cricles). The best-fit parameter estimates are 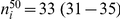 fluorescence units and 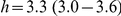. The other parameters employed are listed in Table 1. Initial conditions were the same as in Fig. 2. Inset (A) shows the normalized distribution of CLDN1 expression on mock infected cells (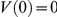) at all post-infection times (lines overlap and are indistinguishable). Inset (B) shows the residual (symbols) of the best-fit to the experimental data; the mean error is 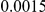 and is not significantly different from zero (line) (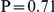 using the two tailed t-test).

We found that following the onset of infection the overall CLDN1 expression decreased with time, as observed by Liu *et al.* [16] (Fig. 3). As a control, no change in the distribution was observed in mock infected cells. Remarkably, our model provided good fits to the measured distribution of CLDN1 expression on cells at day 5 post-infection when the parameters 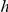 and 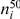, which characterize the Hill function defining the susceptibility, were adjustable. Note that no down-modulation of CLDN1 by HCV was assumed. Cell subpopulations with high CLDN1 expression reached a plateau by day 5 (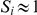 in Fig. 2D), whereas subpopulations with low CLDN1 expression continued to proliferate (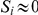 in Fig. 2D). Consequently, the latter cells dominated the culture, explaining the shift in the CLDN1 distribution towards lower mean CLDN1 expression with time (Fig. 3). This drop in the mean CLDN1 expression also explains the observed resistance of the cells in culture at day 5 post-infection to HCV pseudo-particle (HCVpp) entry [16].

### Expression of CLDN1 on uninfected and infected cells

**Figure 4:**
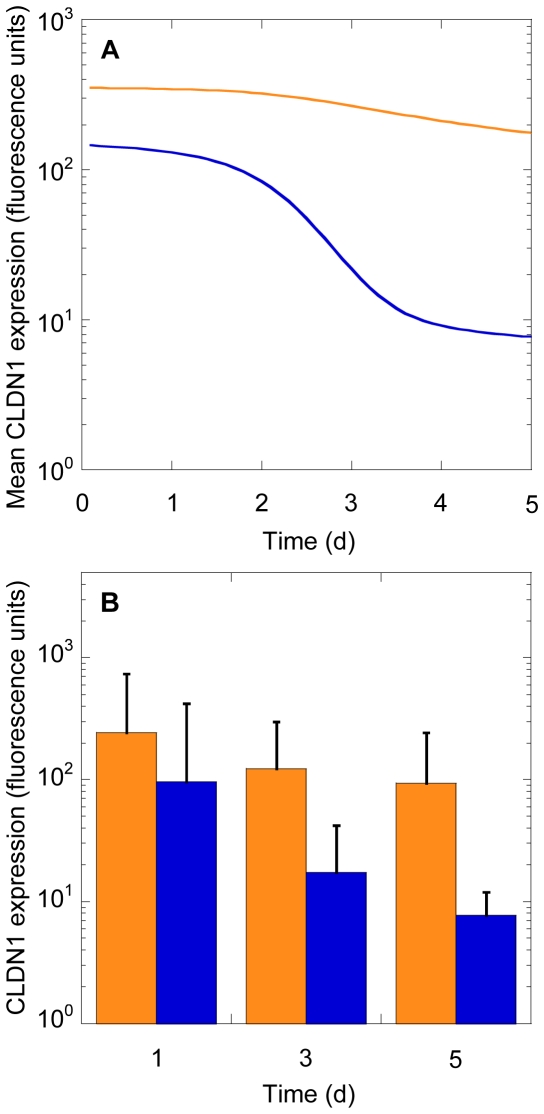
Model predictions of CLDN1 expression on infected and uninfected cells. (A) Time-evolution of the mean CLDN1 expression level on infected (orange) and uninfected (blue) cells. (B) Average CLDN1 expression on twenty cells sampled from the infected (orange) and uninfected (blue) populations at different post-infection times. Error bars represent standard deviations. Parameters and initial conditions employed were the same as in Fig. 2.

We found that the mean CLDN1 expression was greater on infected cells than uninfected cells at any time post-infection (Fig. 4A). This followed from the higher susceptibility to infection of cells expressing more CLDN1. To mimic the observations of Reynolds *et al.* [15], we randomly sampled twenty cells from the infected and uninfected cell populations at different times post-infection. We found that the average CLDN1 expression on the infected cells sampled was higher than on the uninfected cells sampled at all post-infection times considered (Fig. 4B), in qualitative agreement with the observations of Reynolds *et al.* [15] who reported data at day 3 post-infection. The difference between the mean expression level of the samples became significant with the progression of the infection (P = 0.14 at day 1 and P = 0.01 at day 3, using the one-tailed unequal variance Students t-test on the data in Fig. 4B). Again, no active modulation of CLDN1 expression by HCV was assumed. That subpopulations with higher CLDN1 expression were more susceptible and hence preferentially infected underlies the observed higher CLDN1 expression on infected cells than uninfected cells.

## Discussion

Several recent *in vitro* studies have observed superinfection exclusion with HCV [16]–[18], the mechanistic origins of which are yet to be established. The superinfection block has been argued to occur at the level of RNA replication and not at the level of virus entry [17], [18]. The block may also be due to genetic bottlenecks associated with mitosis (Webster B, Wissing S, Herker E, Ott M, and Greene W, presented at the 18^th^ International Symposium on Hepatitis C Virus and Related Viruses, Seattle, Washington, September 2011). More recently, Liu *et al.* [16] found that the mean CLDN1 expression level on cells in culture decreased following HCV infection suggesting that the superinfection block could be at the level of entry and due to HCV-induced down-modulation of CLDN1. Reynolds *et al.* [15] observed, however, that CLDN1 expression on infected cells was higher than on uninfected cells, suggesting an up-regulation of CLDN1 by HCV. The role of HCV in modulating CLDN1 expression and hence inducing a superinfection block at the level of virus entry thus remained unclear. Here, we showed that the different susceptibilities to infection of cells expressing different levels of CLDN1 and the ensuing viral kinetics may render these conflicting observations two sides of the same coin. Our model of viral kinetics without requiring explicit modulation of CLDN1 expression by HCV fit well the measured distribution of CLDN1 expression on cells following exposure to HCV *in vitro* and also described the observed higher CLDN1 expression on cells infected by HCV than those uninfected. Thus, the HCV superinfection block *in vitro* may not be due to HCV-induced down-modulation of CLDN1.

Although CLDN1 is required for HCV entry, its role in mediating entry remains to be established. Only recently have studies identified its association with CD81 as important for entry [8]–[12]. Consequently, a mechanistic description of how the susceptibility of cells to infection depends on CLDN1 expression remains difficult to construct. Here, we have assumed, based on our earlier model of the dependence of the susceptibility on CD81 expression [37], that the dependence of the susceptibility on CLDN1 expression follows a Hill function (Methods). With this assumption and without requiring explicit modulation of CLDN1 expression by HCV, our model fit the observed distribution of CLDN1 expression at day 5 post-infection, giving us confidence in our model. Yet, we cannot rule HCV-induced down-modulation of CLDN1 out entirely, for it is conceivable that some down-modulation of CLDN1 expression by HCV along with a different set of parameters that define the Hill function above would also fit the observed distribution equally well. Indeed, Liu *et al.* showed that coexpression of CLDN1 with the HCV proteins Core and E1E2 in 293T cells inhibited CLDN1 expression [16]. The relative extents of the explicit modulation of CLDN1 by HCV and the shift in the distribution of CLDN1 expression due to the underlying viral kinetics remain to be delineated fully. Infection assays with growth-arrested cells [40], [41] may facilitate this delineation. The distribution of CLDN1 expression (or any entry receptor) would remain unchanged post-infection on such cells if there were no explicit modulation by HCV over times short compared to the average life span of infected cells.

Our model assumed that CLDN1 alone limited HCV entry. However, other receptors may also limit HCV entry. Indeed, in the experimental distribution of CLDN1 expression at day 5 post infection (Fig. 3), a tiny peak appears in the high CLDN1 region possibly because here other entry receptors limit entry. Our model thus did not capture this small peak. Our model was also limited to a qualitative comparison with the experiments of Reynolds *et al.* [15] because the distribution of CLDN1 expression on the Huh-7.5 cell line they used was not known and a quantitative relationship between measured fluorescence intensities and CLDN1 expression remains to be established. Our aim was to present a reconciliation of the disparities in the experimental observations of the modulation of CLDN1 expression following exposure to HCV, which our study accomplishes despite the above limitations.

Importantly, our study points to the significance of considering the underlying viral kinetics in interpreting data of receptor expression following HCV infection. Several studies have argued that HCV entry factors are down-modulated in HCV infected cells. For instance, Liu *et al.* [16] have suggested that not only CLDN1 but also occludin expression levels decreased post-infection in their experiments. Sainz *et al.* [42] claimed down-modulation of Niemann-Pick C1-like 1 (NPC1L1), a recently identified HCV entry factor, following HCV infection. Our study suggests that the observed reduction in the levels of entry receptors could be due to the underlying viral kinetics and not necessarily HCV-induced down-modulation of the receptors. Indeed, long-term HCV infection in cell culture showed that the mean CD81 expression declined as CD81-low cells refractory to infection outgrew CD81-high cells susceptible to infection [17], [38] and the cells infected allowed unhindered subsequent HCVpp entry suggesting minimal HCV-induced down-modulation of CD81 [17]. Thus, whether HCV actively down-modulates entry receptors remains to be established.

## Methods

### Initial CLDN1 expression on cells

We fit the distribution of CLDN1 expression on Huh7 cells [16] using a mixture of two log-normal distributions, given by 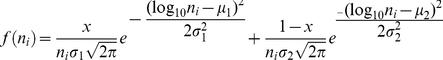, where 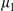 and 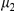 were the means and 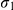 and 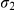 the standard deviations of 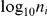, and 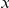 was the mixture coefficient (Fig. 1). We performed the fit using the nonlinear regression tool NLINFIT in MATLAB and examined the goodness of fit by evaluating residuals and characterizing them using the two-tailed t-test.

### Dependence of the susceptibility on CLDN1 expression

Using reaction-equilibrium to estimate the mean number of E2-CD81 complexes formed across a virus-cell contact and a Poisson process to account for stochastic fluctuations, we previously deduced the susceptibility of cells to infection, 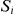, to be a sigmoidal function of CD81 expression [37]. CLDN1 is thought not to interact directly with E2 but to mediate entry through its association with CD81. Accordingly, we retained the sigmoidal form and represented the dependence of 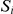 on CLDN1 expression using a Hill function, 
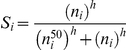
, where 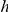 is the Hill coefficient and 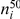 is the CLDN1 expression level at which 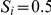. Note that 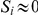 when 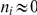 and 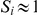 when 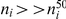.

### Model of HCV viral kinetics

We constructed a mathematical model of HCV kinetics *in vitro*, where the susceptibility of a cell to infection was a function of its CLDN1 expression level. Mimicking experiments, we considered a population of target cells, 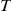, with a known distribution of the CLDN1 expression level across cells exposed to HCVcc virions. We divided the cells into different subpopulations 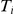 with distinct CLDN1 expression levels 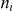, where 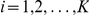. We let 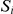 be the relative susceptibility of cells 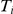 to infection. Cells 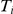 were assumed to proliferate with the rate constant 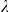, die with the rate constant 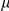, and be infected with the second order rate constant 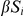, where 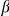 represents the infection rate constant of cells expressing excess CLDN1 (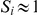). The resulting infected cells, 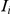, were lost with the enhanced rate constant 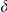 due to HCV-induced cytopathicity [38], [39]. We neglected the proliferation of 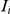 as HCV induces cell cycle arrest [39], [43]. Free virions, 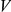, were produced by 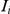 at the rate 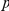 per cell and were cleared with the rate constant 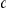. Here, 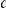 represents the combined rate of the natural degradation of virions, the loss of viral infectivity, and the loss of virions due to entry and attachment [44], [45]. The following coupled dynamical equations then predicted the time-evolution of 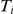, 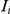, and 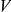 [37]:
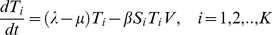
(1)

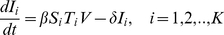
(2)

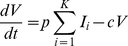
(3)


### Parameters

We solved the above equations using the initial distribution of CLDN1 expression on Huh7 cells measured experimentally (Fig. 1) [16]. We set the initial cell subpopulations to 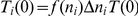, where 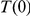 was the total initial target cell population and 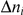 was the narrow range of CLDN1 expression that constituted each subpopulation. We let 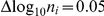 and 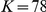; finer discretisation did not alter our results [37]. We allowed 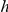 and 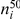 to be adjustable parameters and set the other model parameters (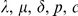 and 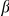) to values that captured the dynamics of infection of Huh-7.5 cells with the JFH-1 strain [37]. Model parameters are summarized in Table 1.

### Data and comparisons with model predictions

We considered data from recently published studies on the modulation of CLDN1 expression following HCVcc infection [15], [16]. First, we considered the experiments of Liu *et al.* [16], where Huh-7 cells were mock infected or exposed to JFH1 virions (0.1 MOI) for 5 days. The total cellular CLDN1 expression level was measured by western blotting and the cell surface expression level was measured by flow cytometry (Fig. 4A and 4B in [16]). We digitized data using Engauge digitizer. Using our model, we predicted the distribution of CLDN1 expression at any time *t* post-infection as 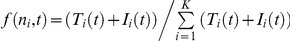. We fit model predictions of the latter distribution at day 5 to the above data using the nonlinear regression tool NLINFIT in MATLAB. We examined the goodness of fit by evaluating residuals and characterizing them using the two-tailed t-test. We also repeated the fits with different initial guesses of the adjustable parameters and found that the fits remained unaltered.

Next, we considered the data of Reynolds *et al.* [15], where Huh-7.5 cells were infected with JFH-1 virions and the average CLDN1 expression on twenty cells from NS5A positive infected, NS5A negative infected and uninfected populations were found at day 3 post-infection (Fig. 6F in [15]). To mimic these observations, we randomly sampled twenty cells each from the infected and uninfected cells present at any time 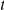 and obtained the corresponding mean and standard deviation of the CLDN1 expression levels on the sampled cells. The probability of choosing a cell 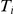 from the population of uninfected cells was 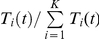 and that of choosing a cell 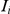 from the population of infected cells was 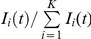. We also computed the time-evolution of the mean CLDN1 expression on all uninfected and infected cells present in culture as 
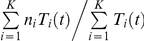
 and 
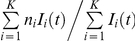
, respectively.
